# PET Imaging Expedites Detection of Aberration in the Humanization of an Annexin A1 Targeting Antibody

**DOI:** 10.3390/ph18030295

**Published:** 2025-02-21

**Authors:** Hailey A. Houson, Brian D. Wright, Solana R. Fernandez, Tim Buss, Sharon L. White, Brittany Cederstrom, James M. Omweri, Jonathan E. McConathy, Jan E. Schnitzer, Suzanne E. Lapi

**Affiliations:** 1 Department of Radiology, University of Alabama at Birmingham, Birmingham, AL 35294, USA; 2 Proteogenomics Research Institute for Systems Medicine (PRISM), La Jolla, CA 92037, USA; 3 Institute of Engineering in Medicine, University of California San Diego, La Jolla, CA 92093, USA

**Keywords:** zirconium-89, antibody imaging, dosimetry, translation

## Abstract

**Objectives**: Annexin-A1 is a 37 kDa phospholipid-binding protein which is concentrated in a truncated 34 kDa form (AnnA1) in caveolae on the tumor vascular endothelial cell surface with expression in many tumor types. PRISM developed the monoclonal mouse antibody mAnnA1 against AnnA1 for evaluation of AnnA1 as a potential target for imaging and therapy in oncology. mAnnA1 was humanized to make hAnnA1 for translation to clinical studies. Both PRISM-produced mAnnA1 and cGMP contractor-produced hAnnA1 were investigated using noninvasive PET/CT imaging, and dosimetry was evaluated to enable clinical translation of this strategy and to investigate in vivo behavior of hAnnA1. **Methods**: Antibodies mAnnA1 and hAnnA1 (PRISM “hAnnA1-P” or contractor generated “hAnnA1-C”) were conjugated with the chelator deferoxamine and evaluated for immunoreactivity with ELISA. Conjugated antibodies were radiolabeled with zirconium-89. Naïve mice, rats, and non-human primates (NHP) were injected with [^89^Zr]mAnnA1 or [^89^Zr]hAnnA1 and imaged with PET/CT up to 10 days post injection. After imaging, mice and rats were euthanized and organs were collected, weighed, and radioactivity was quantified using a gamma counter. Dosimetry in mice and NHPs were calculated using OLINDA. **Results**: [^89^Zr]mAnnA1 showed similar biodistribution to other antibodies with slow clearance through the liver. Transition to [^89^Zr]hAnnA1-C during the dosimetry studies revealed substantial uptake in the spleen (130 ± 48% ID/g at day 5 post injection in female BALB/c), which was not observed with [^89^Zr]mAnnA1 (5.6 ± 1.7% ID/g at day 7 PI). Further studies in multiple strains of mice showed variable elevated splenic uptake of [^89^Zr]hAnnA1-C across mouse strains, with the highest uptake observed in female BALB/c mice (118.4 ± 23.1% ID/g) and the lowest uptake observed in male CD1 mice (34.7 ± 10.2% ID/g). Additionally, splenic uptake of hAnnA1-C was observed in Fischer rats (2.8 ± 0.6% ID/organ) and NHPs (1.6 ± 0.6% ID/organ), although at lower levels than what was observed in BALB/c mice (8.8 ± 1.8% ID/organ). Dosimetry results showed similar values between estimates based on mouse and NHP data, with the largest difference seen in the spleen (5.2 vs. 2.6 mSv/MBq in females respectively). Sequencing of hAnnA1-C revealed a frameshift mutation in the antibody sequence introduced during cGMP manufacture. Restoration of the antibody sequence by PRISM returned the antibody distribution into alignment with mAnnA1. **Conclusions**: An aberration introduced during cGMP production of hAnnA1-C resulted in increased splenic uptake and alteration of the biodistribution in mice. PET imaging enabled quantitative detection of the immunogenic behavior of hAnnA1, which led to detection of the sequence error. Restoration of the sequence resulted in an antibody which was non-immunogenic to mice.

## 1. Introduction

The ability to deliver drugs to cancer cells for imaging and therapy has greatly advanced over the last decades. Highly specific small molecules and antibodies have been developed to bind tumor-associated receptors. Unfortunately, a limitation of the current delivery paradigm is that drugs which are delivered orally or intravenously must cross the vascular endothelium in order to reach their tumor-associated target. The requirement to cross the vascular barrier is often a major hindrance to drug delivery and significantly impedes drug delivery to the target [1]. This has led to the therapeutic strategy of over-delivering therapeutics to the blood to drive the blood-to-tissue concentration gradient in order to achieve therapeutic drug concentrations in the tumor [2]. Dose-limiting toxicities frequently ensue.

With this in mind, the Proteogenomics Research Institute for Systems Medicine (PRISM) investigated the proteomic map of tumor endothelium and discovered a potential target, Annexin A1 (37 kDa), which is overexpressed on tumor endothelial caveolae [3]. Annexin A1 is a phospholipid binding protein which has been studied as a downstream mediator of glucocorticoid function [4]. Further investigation revealed that the annexin A1 in the tumor endothelium exists as a truncated (34 kDa) form which is potentially induced by the tumor environment [3]. Annexin A1 has been observed to be overexpressed by several cancer types including lung, pancreatic, liver, and colorectal cancers [5,6,7,8]. Additionally, high expression of annexin A1 has been associated with worse patient outcomes. Additional work has shown caveolae targeting greatly improves the therapeutic efficacy of other antibodies [9].

Positron emission tomography (PET) allows for noninvasive and quantitative tracking of injected radiopharmaceuticals. Zirconium-89 (t_1/2_ = 3.2 days) is a positron-emitting radioisotope which can be used for PET imaging up to several days post-injection. Attaching zirconium-89 to an antibody allows for PET imaging for detection and quantification of the antibody for multiple days post-injection. Zirconium-89 labeled antibodies have been used both preclinically and in clinical trials to identity tumor receptor expression and perform dosimetry [10,11].

A significant strength of PET imaging in drug development is that it allows the investigator to quantitatively track the location of the radiotracer in the body over time. This type of preclinical evaluation can be used to predict appropriate cancer models, doses needed to achieve response, and perform dosimetry evaluation before the clinical translation of a drug [12,13].

The mouse AnnexinA1 antibody mAnnA1 was humanized for clinical translation into hAnnA1. In this paper we describe how PET imaging of the pharmacokinetic properties of zirconium-89-labeled mAnnA1 and hAnnA1 revealed a sequence error during cGMP production of hAnnA1.

## 2. Results

Antibodies were investigated for immunoreactivity after DFO conjugation and did not display reduction in immunoreactivity (Figure S1).

**Figure 1:**
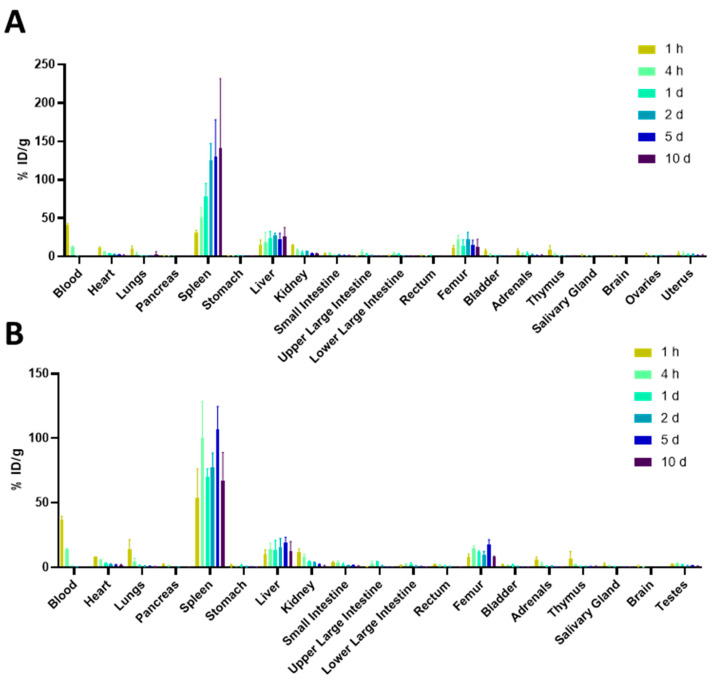
Dosimetry of [^89^Zr]hAnnA1-C in BALB/c mice. Mice were injected with 25 μg of [^89^Zr]hAnnA1-C. At each timepoint, organs from female (**A**) and male (**B**) mice (*n* = 4/group) were harvested for biodistribution analysis.

Dosimetry in male and female BALB/c mice was investigated with the humanized annexin A1 binding antibody [^89^Zr]hAnnA1-C (Figure 1). At each timepoint following [^89^Zr]hAnnA1-C injection, four male and four female mice were euthanized and their organs were collected for weighting and gamma counting. At 10-day post injection, splenic uptake was 141.4 ± 90.2% ID/g in females and 67.1 ± 21.6% ID/g in males. Additionally, hAnnA1-C was more rapidly cleared from the blood than mAnnA1, with 0.08 ± 0.03% ID/g in female mice at 5-day PI, whereas at 7-day post-injection the level of mAnnA1 in the blood was 6.18 ± 1.34% ID/g.

It did not appear that variability in organ mass was a significant contributor to % ID/g values observed (Figure S4). While spleen mass changed slightly throughout the study (Figure S4C), it did not account for the biodistribution values, which were in significant excess compared to what was observed with mAnnA1 (10.2 ± 6.9% ID/g at 7D PI) in BALB/c mice (Figure S2).

**Figure 2:**
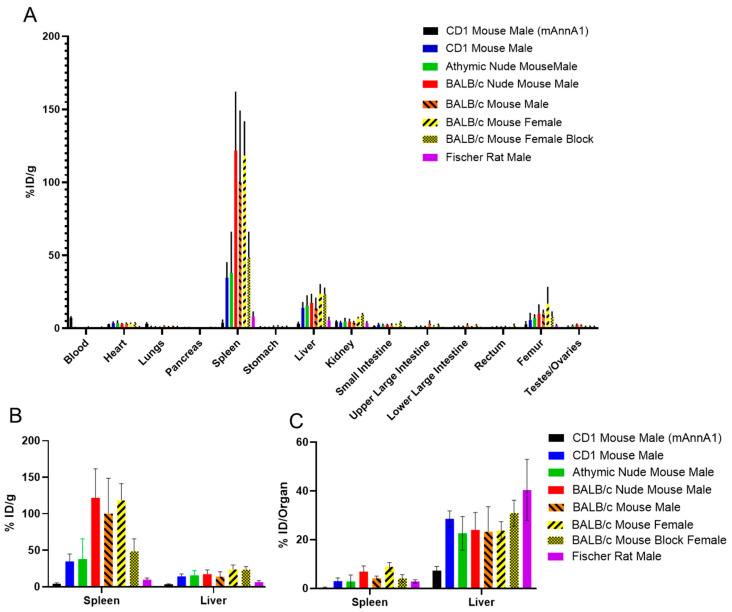
Biodistribution of mAnnA1 and hAnnA1-C varies across strains of lab mice. Biodistribution of [^89^Zr]mAnnA1 and [^89^Zr]hAnnA1-C at 48 h post injection was investigated in male CD1 mice (*n* = 6), [^89^Zr]hAnnA1-C was investigated in male athymic nude mice (*n* = 4), male BALB/c nude mice (*n* = 6), male wildtype BALB/c mice (*n* = 6), and female wildtype Balb/c mice (*n* = 6) injected with 25 μg of hAnnA1-C. Mice in the wildtype BALB/c blocking group were injected with 750 μg hAnnA1-C (*n* = 4), and Fischer rats were injected with 100 μg of hAnnA1-C (*n* = 4). Male CD1 mice dosed with [^89^Zr]mAnnA1 (*n* = 4) were injected with 25 μg. Whole body distribution represented as % ID/g (**A**), and extracted spleen and liver uptake (**B**) are shown along with % ID/Organ (**C**).

Biodistribution studies were performed at 48 h post injection in several common lab strains of mice and in Fischer rats (Figure 2). A significantly different biodistribution pattern was observed with the humanized [^89^Zr]hAnnA1-C as compared to the murine [^89^Zr]mAnnA1. In a direct comparison between mAnnA1 and hAnnA1-C in male CD1 mice, we observed lower splenic (4.1 ± 1.5% ID/g) and liver (3.2 ± 0.9% ID/g) uptake of mAnnA1 as compared to the splenic (34.8 ± 10.2% ID/g) and liver (14.2 ± 3.5% ID/g) uptake observed with hAnnA1-C. Blood clearance was also much faster with hAnnA1-C, with concentrations of 0.3 ± 0.3% ID/g observed at 48 h post injection compared to 7.3 ± 0.7% ID/g when using mAnnA1. Across the mouse strains investigated, the highest splenic uptake was observed in BALB/c strains as compared to CD1 and athymic nude mice. Additionally, there was no difference in splenic uptake between the BALB/c nude mice, male BALB/c, and female BALB/c mice (Figure S3). The high splenic uptake was observed to be at least partially saturable, as we observed a decrease in uptake in female BALB/c mice to 48.5 ± 17.7 from 118.4 ± 23.1% ID/g when co-injected with a 750 μg cold dose of hAnnA1-C (Figure 2B). The Fischer rats appeared to have less splenic uptake when comparing % ID/g values; however, because rats are larger than mice, representation as % ID/organ may be more appropriate when comparing uptake across species. Splenic uptake in Fischer rats (2.8 ± 0.6) was comparable to athymic nude (2.8 ± 2.6) and CD1 (2.9 ± 1.4) mice when expressed at % ID/organ (Figure 2C). Further information on the results in Fischer rats is provided in Figure S5. Liver uptake was very similar across all mouse strains and Fischer rats.

**Figure 3:**
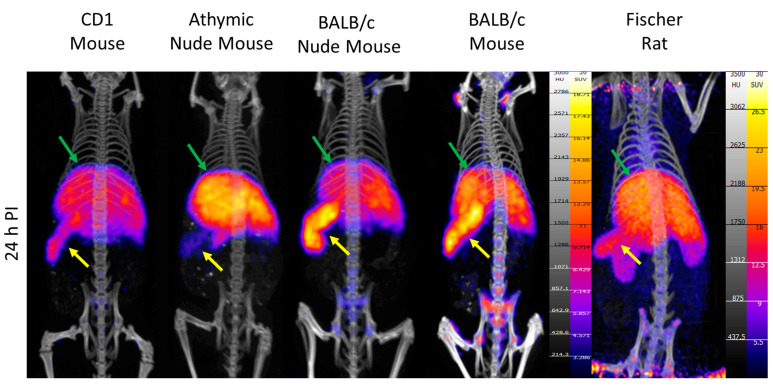
PET/CT images of mice and rats injected with [^89^Zr]hAnnA1-C. The spleen and liver are denoted with yellow and green arrows, respectively.

In PET/CT images, the difference in spleen uptake of [^89^Zr]hAnnA1-C between the mice strains is visibly apparent (Figure 3). Both the BALB/c and BALB/c nude mice have higher splenic uptake than the CD1 and athymic nude mice, which is visible in the PET/CT images (Figure 3). Liver uptake appears similar between all the mice strains and Fischer rats. Very little uptake is visible in any other organ, except some bone uptake, which is attributable to free zirconium-89, as has been described in other mAb studies [14].

**Figure 4:**
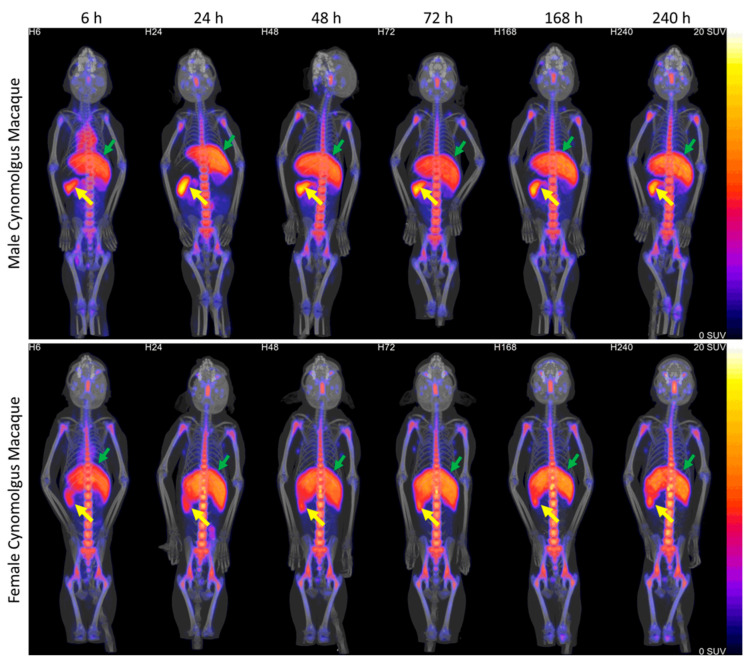
Biodistribution of [^89^Zr]hAnnA1-C in NHPs. Male (*n* = 1) and female (*n* = 2) cynomolgus macaques were imaged from 6 h to 240 h (10 days) post injection of [^89^Zr]hAnnA1-C (0.3 mg/kg, 1–2 mCi). Spleen and liver are denoted with yellow and green arrows respectively.

Radiolabeling of [^89^Zr]hAnnA1-C at Charles River Mattawan was performed according to procedures developed at UAB, and with DFO-hAnnA1-C and zirconium-89 provided by UAB. After injection, NHPs were imaged using PET/CT out to 6 days. Early observation showed that the majority of [^89^Zr]hAnnA1-C in NHPs was cleared from the circulation after 24 h, which was noted by the clearance of radioactivity in the heart (Figure 4). Spleen and liver uptake was apparent at all time points, along with bone in the spinal column and the ends of long bones. After the initial imaging timepoint at 6 h, there was very little change apparent in the PET/CT images. One male NHP died during the 24 h imaging timepoint due to aspiration pneumonia.

**Figure 5:**
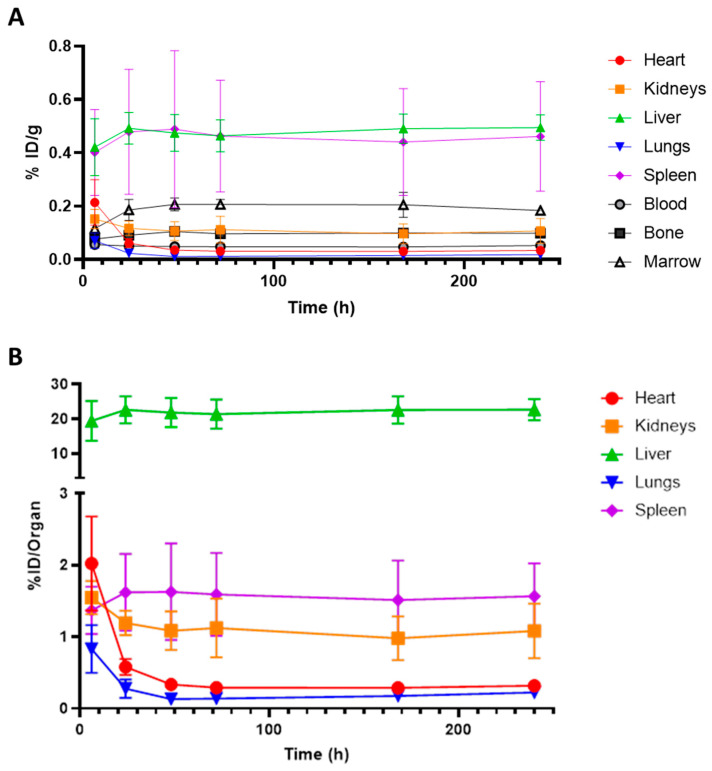
Quantification of NHP PET/CT imaging results. SUV analysis of the PET/CT images was performed to produce % ID/g (**A**) values through the imaging timepoints. Conversion of % ID/g to % ID/organ (**B**) was performed using estimated organ masses from published distribution data [15].

Uptake of [^89^Zr]hAnnA1-C was observed to be highest in spleen and liver at all observed datapoints for the NHP data extracted as % ID/g (Figure 5A). When converted to % ID/organ, spleen uptake appeared to be similar to uptake in kidneys (Figure 5B). Clearance from circulation was observed in the decrease of radioactivity in the heart; however, activity was retained over the time studied in the liver, spleen, and kidneys.

Data from the mouse and NHP dosimetry studies was used to predict human dosimetry of [^89^Zr]hAnnA1-C (Table 1). From the mouse dosimetry study, the organs which were predicted to receive the highest dose were the spleen, liver, adrenals, gallbladder wall, and kidneys (4.7, 2.9, 1.4, 1.4, and 1.2 mSv/MBq in males, respectively). Estimated dose was higher in females than in males, and was driven primarily by increases in kidney, liver, and spleen uptake.

Dose estimation from NHP data shows the organs with the highest dose predicted as the liver, spleen, adrenals, gallbladder wall, and kidneys (3.0, 1.7, 1.2, 1.2, and 1.0 mGy/MBq in females, respectively). The difference in dose between males and females was less apparent in NHPs than in mice.

**Figure 6:**
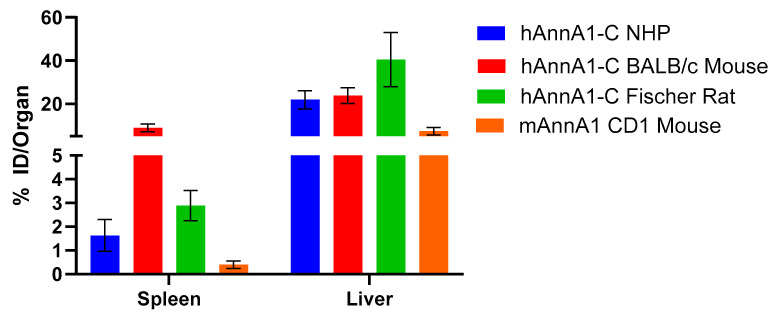
Spleen and liver comparisons between different model organisms. Spleen and liver uptake expressed as % ID/organ was compared between NHPs, mice, and rats. Additionally, uptake of mAnnA1 in CD1 mice at the same timepoint was provided as a comparison.

Of the strains and species investigated, the highest [^89^Zr]hAnnA1-C uptake was observed in the spleens of BALB/c mice (8.8 ± 1.8% ID/organ), followed by rats (2.9 ± 0.6% ID/organ) and NHPs (1.6 ± 0.7% ID/organ) (Figure 6). Splenic uptake of [^89^Zr]hAnnA1-C was higher in all models than uptake observed with mAnnA1 in mice (0.4 ± 0.2% ID/organ) (Figure 6).

**Figure 7:**
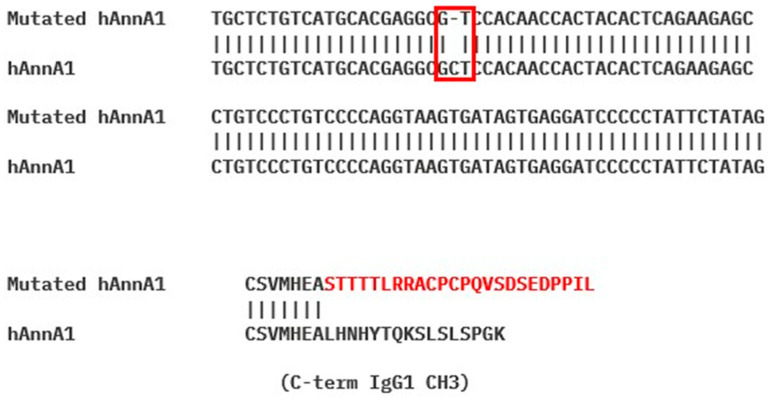
Discovery of frameshift mutation in hAnnA1-C. Single nucleotide deletion at position 1423 (red box) was discovered after production of the cGMP lot. The resulting frameshift mutation occurring in the heavy chain of the antibody altered 16 amino acids and added 8 more amino acids than the intended antibody marked in red.

In the late stage of the production of the cGMP lot of hAnnA1-C, it was discovered that the contractor’s expression plasmid contained a nucleotide deletion resulting in a frameshift mutation in the translated sequence. This mutation resulted in 16 altered amino acids and 8 additional amino acids than the antibody was not intended to contain (Figure 7). The mutation in the antibody sequence was not discovered until the cGMP lot was vialed and stability studies were underway, and after all data from previous Figure 1, Figure 2, Figure 3, Figure 4, Figure 5 and Figure 6 had been collected. This paper will refer to the mutated sequence antibody as hAnnA1-C (contractor-generated) and the correct sequence antibody as hAnnA1-P (PRISM-generated).

**Figure 8:**
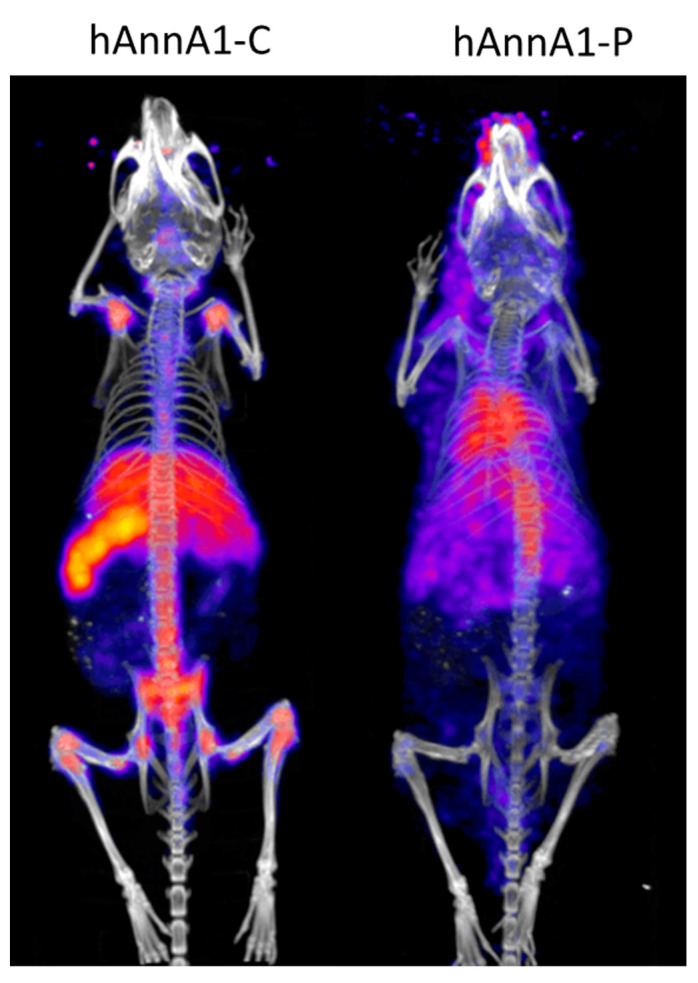
Comparison of frameshift mutant hAnnA1-C and correct sequence hAnnA1-P. Mice were imaged using PET/CT 24 h after injection of 25 μg of either [^89^Zr]hAnnA1-C or [^89^Zr]hAnnA1-P.

The in vivo behavior of hAnnA1-C and hAnnA1-P antibodies was evaluated 24 h after injection in male CD1 mice (Figure 8). Visual assessment of the antibody distribution showed that the hAnnA1-C cleared from the blood and was concentrated into the liver, spleen, and bone. hAnnA1-P, however, remained in the blood, as shown by the visible cardiac blood pool and diffuse uptake through the body.

**Figure 9:**
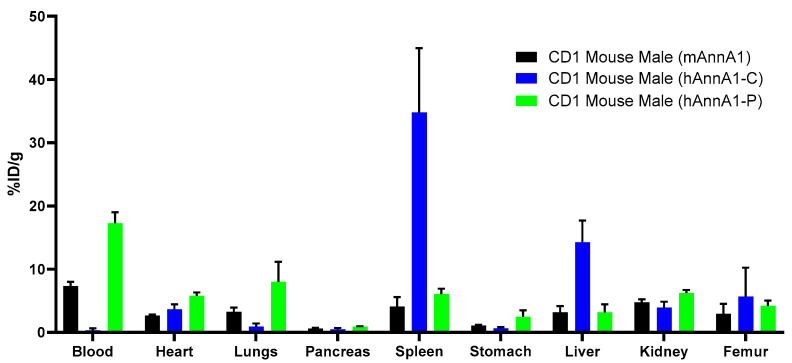
hAnnA1-P behaves similarly to mAnnA1 in mice. Biodistribution comparison of [^89^Zr]mAnnA1, [^89^Zr]hAnnA1-C, and [^89^Zr]hAnnA1-P in mice 48 h post injection.

Biodistribution of male CD1 mice 48 h after injection showed that mAnnA1 was similar to hAnnA1-P in terms of blood, spleen, and liver levels (Figure 9). hAnnA1-C showed rapid clearance from the blood and high uptake in the spleen and liver, as was previously observed.

## 3. Discussion

Development of monoclonal antibodies as therapeutics has grown rapidly, with over 120 approved mAb therapies approved in the US as of 2022 [16]. Monoclonal antibodies show high specificity for their molecular targets and exhibit a high and sustained concentration in the blood. However, a significant challenge in the clinical translation of mAbs is the demonstration of immunogenic responses by the host organism [17,18]. Immunogenic reactions can range from an immediate acute histamine response to delayed hypersensitivity [19].

One of the most important factors impacting the immunogenicity of an antibody is the glycosylation pattern on the fragment crystallizable region of the antibody [20]. Even in well controlled production conditions, the glycosylation of proteins can vary significantly [21]. Previously, the monoclonal IgG1 trastuzumab has been observed to be less effective (antibody-dependent call-mediated cytotoxicity) in lots which contain less fucosylation, and more effective in lots containing more mannose [22,23].

Additionally, variation in the genetic background of the model animal is known to alter biodistribution [24]. High splenic uptake has been observed in mouse strains with a profound degree of immunodeficiency, which can be reduced by removing glycosylation from the antibody [24,25]. However, in “moderately” immunodeficient mice, such as Nu/Nu mice, and immunocompetent mice, such as CD1 and BALB/c mice, this has not been widely observed [24]. This accumulation in the spleen appeared to accelerate clearance from the blood in NSG mice as compared to NOD SCID mice. Additionally, when the glycosylation was removed from the antibodies, this splenic uptake was observed to decrease [25].

Studies using mAnnA1 in mice showed levels of splenic uptake consistent with the commercially available humanized antibody trastuzumab (5.6 ± 1.7 and 7.2 ± 2.2% ID/organ respectively at 7-day post injection Figure S2). PET imaging studies using the cGMP produced hAnnA1 (hAnnA1-C) immediately showed that the distribution of the antibody had changed dramatically, despite the molecular target being the same. In an effort to understand the reason for this change, we investigated whether the difference was related to mouse strain. The mouse strain which displayed the highest splenic uptake was the BALB/c mouse, which showed high uptake across the immunocompetent and immunodeficient strains. Additionally, we observed that hAnnA1 (hAnnA1-C) showed high splenic uptake in mice, rats, and NHPs which were naïve to the antibody.

Dosimetry studies of hAnnA1-C in mice and NHPs revealed a similar predicted estimated dose in humans. In both models, the organs with highest predicted dose were the liver and spleen. Splenic dose was lower in the NHP dosimetry data compared to the Balb/c dosimetry. This result was predictable, as Balb/c mice had the highest splenic uptake, highlighting the importance of strain selection in dosimetry studies. Other organs which might be dose limiting factors are adrenals, gall bladder, and kidneys. Bone uptake was observed to be lower in mice than in NHPs. Overall, splenic dose was similar between rats and NHPs.

Unfortunately, a sequence mutation was introduced during the production of the cGMP lot of hAnnA1-C by NCI contractors. This error was not discovered until the cGMP batch was vialed and all dosimetry studies were complete. This sequence mutation resulted in the alteration of 16 amino acids and the addition of 8 amino acids to the heavy chain of hAnnA1. Upon this discovery, we compared the distribution of the aberrant antibody hAnna1-C with the correct sequence hAnnA1-P produced by PRISM. The in vivo behavior of hAnnA1-P was very similar to mAnnA1 and did not show rapid blood clearance or high splenic uptake. Unfortunately, it was clear that the vialed cGMP produced hAnnA1 (hAnnA1-C) would not be suitable for translation into humans.

Rapid whole body quantitative PET imaging enabled the detection of the immunogenic mutation in the cGMP lot of hAnnA1 before translation into humans. We confirmed that the apparent immunogenicity was not limited to rodents but was replicated in nonhuman primates. Future work will involve the manufacture of hAnnA1 without sequence errors, dosimetry study in mice, and evaluation of toxicity in rodents in preparation for translation into humans.

## 4. Materials and Methods

All reagents were purchased from Fisher Scientific (Hampton, NH, USA) unless otherwise stated.

mAnnA1 was generated by PRISM (La Jolla, CA, USA). The mouse monoclonal antibody, mAnnA1 [3], was generated using conventional hybridoma methodology [26]. To humanize the antibody, the complementarity determining regions (CDRs) were grafted onto the framework of an appropriate germline human antibody [27,28,29]. A single framework back mutation was introduced in both the heavy chain and light chain to restore affinity to the same level as mAnnA1. The humanized antibody sequence was provided to contractors for the NCI NExT program for production of a cGMP lot which was used in this study (designated hAnnA1-C).

Deferoxamine thioscyanate (p-SCN-Bn-DFO) was purchased from Macrocyclics (Plano, TX, USA). Conjugation of mAnnA1 or hAnnA1 was performed as previously described [30]. Antibodies (>5 mg/mL) were buffer exchanged into 0.1 M sodium carbonate buffer pH 9 using a 40 kDa MW Zeba Spin column (0.5 mL). p-SCN-Bn-DFO was dissolved in dimethyl sulfoxide to a concentration of 10 mM and combined with mAnnA1 at a molar ratio of 6:1 and 3:1 or hAnnA1 at a molar ratio of 1:1. Reactions were allowed to incubate at 37 °C for 1–2 h with gentle shaking. Excess DFO was removed by passing through Zeba Spin columns equilibrated in 1 M HEPES buffer pH 7 two times. Antibody concentrations were determined using Pierce BCA assay.

DFO conjugated mAnnA1 (DFO-mAnnA1) and hAnnA1 (DFO-hAnnA1) were radiolabeled with zirconium-89 produced at the University of Alabama at Birmingham (UAB) Cyclotron Facility [31]. Zirconium-89 was provided in 1 M oxalic acid and was neutralized with 2 M NaOH and 1 M HEPES buffer to pH 7.0–7.4. For radiolabeling, DFO-mAnnA1 was combined with zirconium-89 at a specific activity of 10–50 mCi/mg (0.37–1.85 MBq/mg), while DFO-hAnnA1 was combined at a specific activity of 10 mCi/mg (0.37 MBq/mg). Radiolabeling occurred at 37 °C for 1 h with gentle shaking. Radiochemical purity was determined by developing iTLC strips (Agilent, Santa Clara, CA, USA) in 50 mM diethylenetriamine pentaacetate (DTPA). In these conditions, zirconium-89 moved with the solvent front, and antibody-bound zirconium-89 remained at the baseline.

All animal experiments were performed under the approval of the institutional care and use committee of the University of Alabama at Birmingham (APN 22047). Mice and rats were purchased from Charles River (Wilmington, MA, USA) and allowed to acclimate for 5 days before use. Mouse strains used were BALB/c mouse (code 028), BALB/c nude mouse (code 194), CD1 mouse (code 057), and athymic nude mouse (code 490) and the rat strain was Fischer rat (code 403).

Mouse dosimetry was performed by injecting 25 μg [^89^Zr]hAnnA1-C (~100 uCi, 3.7 MBq) into male and female BALB/c mice. At 1 h, 4 h, 24 h, 2 days, 5 days, and 10 days post injection, four male and four female mice were euthanized, and organs were collected, weighed, and counted on a gamma counter (Hidex Gamma Counter, Turku, Finland). Blood was obtained via cardiac puncture, and 50 μL was taken for gamma counting. Percent injected dose per gram (% ID/g) and percent injected dose per organ (% ID/organ) were calculated by dividing the dose in the organ by the total dose injected. The area under the time activity curve for each organ was calculated using a numerical integration method in Excel using the linear-log trapezoidal method. The result in MBq-hr/MBq was entered into the kinetics portion of the OLINDA/EXM program (Hermes Medical, Stockholm, Sweden). Adult human models (ICRP 89 [32]) were used to calculate organ doses and effective doses in humans.

The non-human primate (NHP) study was performed by Charles River Labs-Mattawan (Mattawan, MI, USA). DFO conjugated hAnnA1-C and zirconium-89 were provided by UAB, and radiolabeling and purification was performed on site according to UAB protocols. Cynomolgus macaques aged 24–60 months (two male, two female) were injected with 1–2 mCi (37–74 MBq) of [^89^Zr]hAnnA1-C (0.3 mg/kg). NHPs were imaged at 6 h and 24 h (30 min static PET) and 48 h, 72 h, 168 h, and 240 h (60 min static PET), followed by CT (120 kVp, 4 mA, 4 s). Images were processed using VivoQuant V3 image processing software (Perceprive, Needham, MA, USA) to give percent injected dose per gram (% ID/g) values and dosimetry was calculated using OLINDA/EXM. Values of percent injected dose per organ (% ID/organ) were calculated from % ID/g values, and estimated organ weights were calculated based on the NHP’s age and body weight according to established estimates [15].

An aliquot of the contractor-generated hAnnA1 Master Cell Bank was submitted to SynBuild as part of normal release testing to confirm the transgene sequence. It was noticed that a single nucleotide deletion was present towards the end of the hAnnA1 CH3 domain, which resulted in a frameshift. Sequence corrected hAnnA1 (hAnnA1-P) was produced by PRISM and radiolabeling, and PET imaging was performed at UAB according to the procedures developed for mAnnA1 and hAnnA1-C. For imaging, mice were administered 25 μg [^89^Zr] hAnnA1-P. Mice (*n* = 4) were imaged 24 h post injection, and then organs were harvested for biodistribution analysis.

All data are displayed as mean ± standard deviation.

## 5. Conclusions

During the cGMP manufacture of hAnnA1, an introduced sequence error resulted in the creation of hAnnA1-C. PET/CT imaging revealed that hAnnA1-C showed a drastic alteration in the splenic uptake and blood clearance of the antibody from the mouse mAnnA1. PET imaging revealed the change was most apparent in immunocompetent and immunodeficient BALB/c strains of mice. High splenic uptake was additionally observed in rats and NHPs, although it did not reach the levels observed in the BALB/c mice. PET/CT imaging of the correct antibody sequence using hAnnA1-P showed a very similar distribution to mAnnA1.

## Figures and Tables

**Table 1:** Human dose estimation from [^89^Zr]hAnnA1-C in BALB/c mice and NHPs.

Target Organ	Predicted by Mouse Dosimetry (mSv/MBq)		Predicted by NHP Dosimetry (mGy/MBq)	
	Female	Male	Female	Male
Adrenals	2.08	1.41	1.295	1.28
Brain	0.0623	0.0447	0.246	0.26
Breasts	0.234	N/A	0.269	N/A
Esophagus	0.563	0.449	0.582	0.573
Eyes	0.126	0.0942	0.247	0.261
Gallbladder Wall	0.957	1.38	1.28	1.88
Heart Wall	0.511	0.587	0.56	0.746
Kidneys	1.4	1.2	1.032	1.16
Left colon	0.712	0.612	0.536	0.576
Liver	3.32	2.96	3.09	2.92
Lungs	0.43	0.369	0.486	0.522
Osteogenic Cells	0.247	0.204	0.993	1
Ovaries	0.66	N/A	0.346	N/A
Pancreas	0.943	0.645	0.867	0.728
Prostate	N/A	0.198	N/A	0.344
Rectum	0.256	0.199	0.326	0.373
Red Marrow	0.314	0.259	0.873	0.767
Right colon	0.699	0.834	0.575	0.694
Salivary Glands	0.184	0.206	0.238	0.29
Small Intestine	0.743	0.624	0.56	0.623
Spleen	5.19	4.74	1.745	2.57
Stomach Wall	0.765	0.609	0.535	0.612
Testes	N/A	1.04	N/A	0.249
Thymus	0.384	0.36	0.358	0.41
Thyroid	0.201	0.173	0.275	0.332
Urinary Bladder Wall	0.19	0.166	0.221	0.302
Uterus	0.496	N/A	0.315	N/A

## Data Availability

The original contributions presented in this study are included in the article/Supplementary Materials. Further inquiries can be directed to the corresponding author.

## Supplementary Materials

The following supporting information can be downloaded at: https://www.mdpi.com/article/10.3390/ph18030295/s1, Figure S1: Conjugation and radiolabeling of mAnnA1 and hAnnA1-C; Figure S2: Biodistribution comparison at 7-day post injection; Figure S3: Statistical analysis of the spleen and liver uptake of ^89^Zr-hAnnA1-C in different rodent strains and sexes; Figure S4: Organ masses from mouse dosimetry study; Figure S5: PET/CT imaging and biodistribution in Fischer rats shows similar spleen uptake to mice.
