# Supplementary material for: PET Imaging Expedites Detection of Aberration in the Humanization of an Annexin A1 Targeting Antibody
Source: Pharmaceuticals (Basel). 2025 Feb 21;18(3):295. doi: 10.3390/ph18030295 (PMC11946604; doi:10.3390/ph18030295)
Supplement: Supplementary file 1 [file pharmaceuticals-18-00295-s001.zip › pharmaceuticals-3469125-supplementary.pdf]

Supplement: Methods

Immunoreactivity was assessed using an indirect ELISA to human Annexin A1 protein. Briefly, ~90 ng human Annexin A1 protein (Abcam, Cambridge, UK) in water was added to each well of an ELISA plate (Nunc, Thermo Fisher, Waltham, MA, USA) and evaporated to dryness at 37°C. The next day, additional protein binding sites were blocked using a blocking solution of 1% BSA in 1X PBS with 0.01% Tween-20. Antibodies were added into blocking solution, and a 12-step serial dilution (210–0.002 nM) was performed in blocking solution. After incubation, wells were washed with washing buffer (1X PBS with 0.5 % Tween-20) and a secondary antibody was added: Invitrogen goat anti mouse HRP for mAnnA1 (Thermo Fisher, Waltham, MA, USA), or Southern Biotech mouse anti human-IgG FC-HRP for hAnnA1 (Birmingham, AL, USA). After 1 h incubation, the secondary antibody was washed off and the ELISA was developed using TMB buffer. The reaction was stopped using 1 M sulfuric acid and read on a plate reader at 450 nm.

Supplement: Results

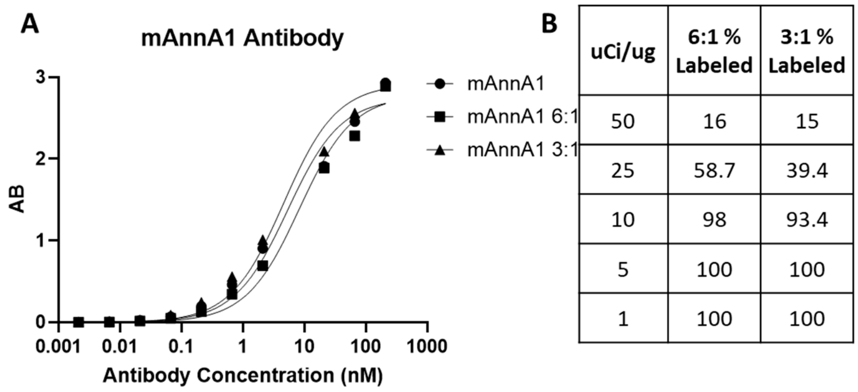

B

uCi/ug	6:1 % Labeled	3:1 % Labeled
50	16	15
25	58.7	39.4
10	98	93.4
5	100	100
1	100	100

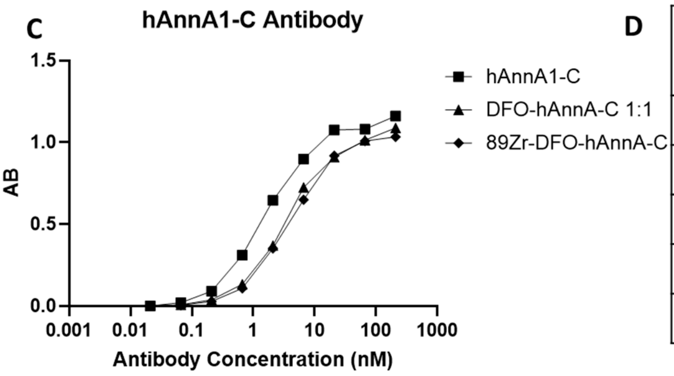

D

uCi/ug	1:1 % Labeled
50	81.5
25	93.1
10	100
5	100
1	100

Figure S1: Conjugation and radiolabeling of mAnnA1 and hAnnA1-C. mAnnA1 antibody was conjugated to DFO at a ratio of 6:1 and 3:1 moles DFO:mAnnA1 (**A**), while hAnnA1-C was conjugated at a molar ratio of 1:1 DFO:hAnnA1-C (**C**). The conjugated DFO-mAnnA1 and DFO-hAnnA1-C were radiolabeled with ^{89}Zr at a range of specific activities (**B,D**).

Conjugation of mAnnA1 with DFO was investigated at a molar ratio of 6:1 and 3:1 moles DFO to mAnnA1. When the material was assessed for immunoreactivity to human Annexin A1 protein with ELISA, both the 6:1 and 3:1 conjugations had retained a significant portion of the binding of the starting mAnnA1 (Figure S1A). When it was assessed for labeling with zirconium-89, both the conjugates labeled above 90% up to 10 mCi/µg (Figure S1B).

Initial test conjugations of hAnnA1-C at 6:1 and 3:1 with DFO revealed a significant drop in the immunoreactivity as compared to unconjugated hAnnA1 (data not shown). A conjugation ratio of 1:1 was observed to retain most of the immunoreactivity of hAnnA1 (Figure S1C). Additionally, radiolabeling was not observed to change the binding affinity of DFO-hAnnA1-C (Figure S1C). When radiolabeled with ^{89}Zr, we observed >90% labeling at 25 µCi/µg (Figure S1D).

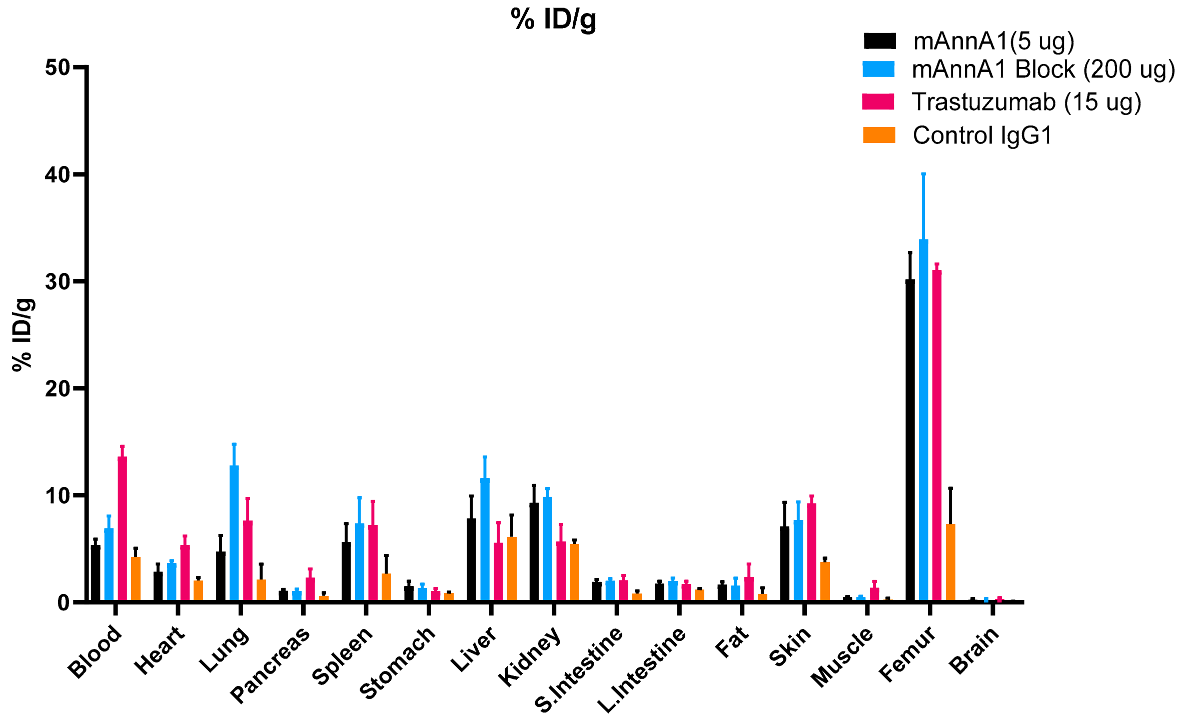

Figure S2 Biodistribution comparison at 7 d post injection. Biodistribution of 5 µg of mAnnA1 in BALB/c mice (n = 10), 200 µg mAnnA1 in BALB/c mice (n = 4), 15 µg of Trastuzumab in BALB/c mice (n = 4), and 5 µg of control IgG in 4T1 bearing BALB/c mice (n = 4).

Biodistribution at 7 d post injection was compared in BALB/c mice using different imaging agents (Figure S2). Trastuzumab was observed to have the highest level in blood with 13.6 ± 0.9 % ID/g, which was higher than all other groups with a $p < 0.001$ (ordinary 2-way ANOVA with Tukey's multiple comparison test). Spleen levels were similar between groups.

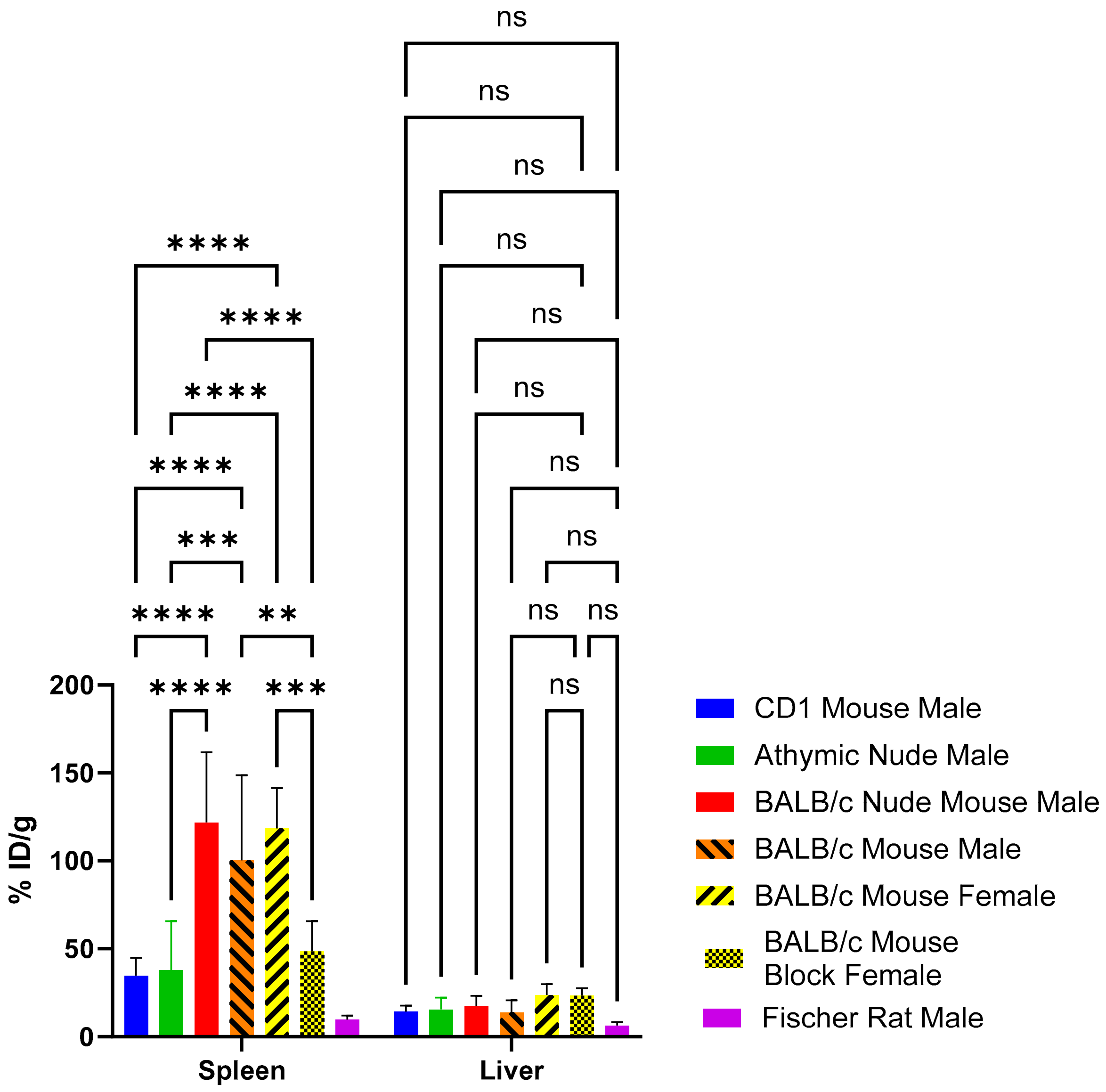

Figure S3: Statistical analysis of the spleen and liver uptake of ^{89}Zr-hAnnA1-C in different rodent strains and sexes.

Statistical comparison between spleen and liver uptake values in different strains of mice and rats was performed using a 2-way ANOVA with a Tukey's multiple comparison test. *p* values are indicated as * $p < 0.05$. ** $p < 0.01$, *** $p < 0.001$, and **** $p < 0.0001$.

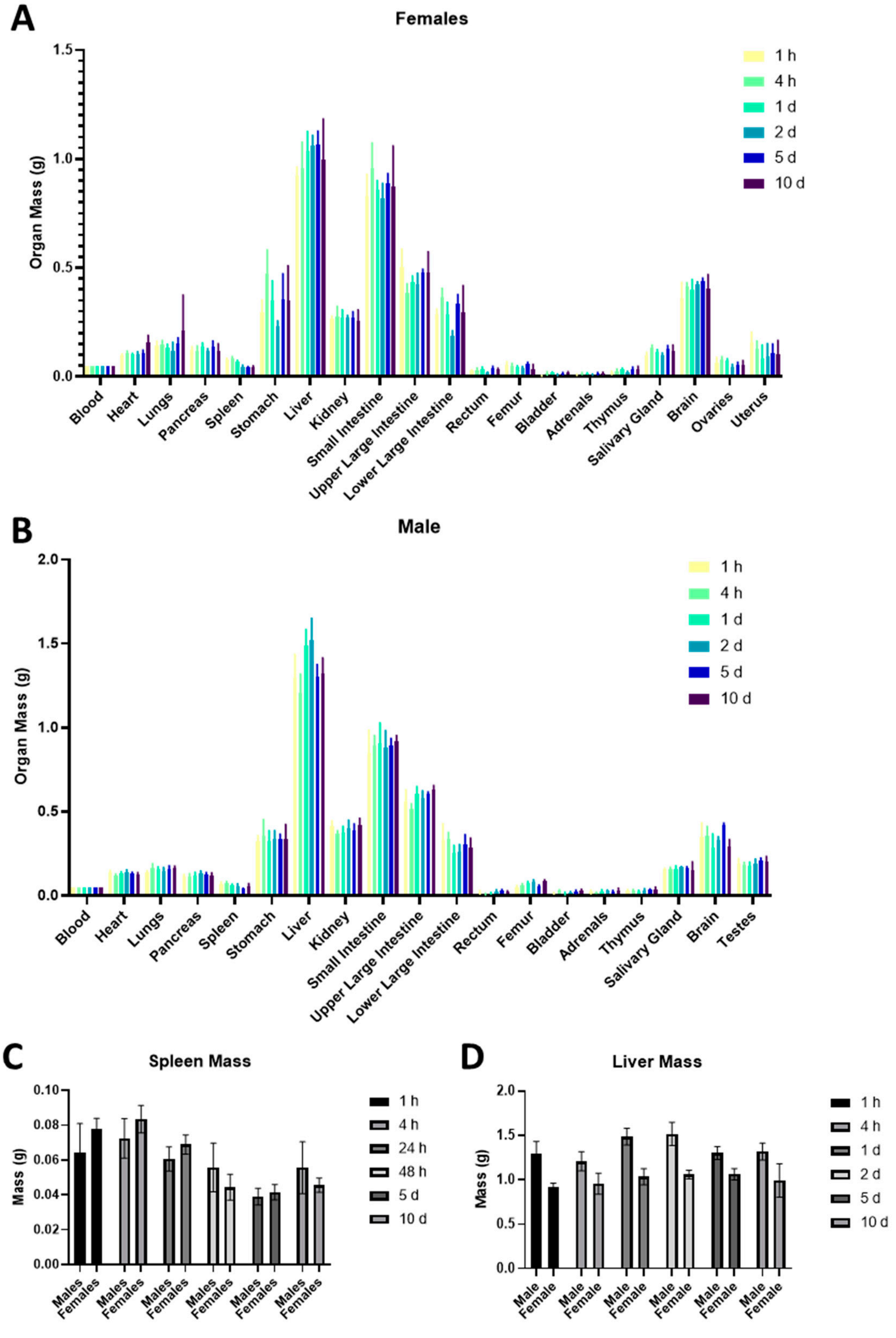

Figure S4: Organ masses from mouse dosimetry study.

Organ masses for each organ were tracked over time in the mouse dosimetry study. A decrease in spleen mass was observed over the study, while liver mass did not change.

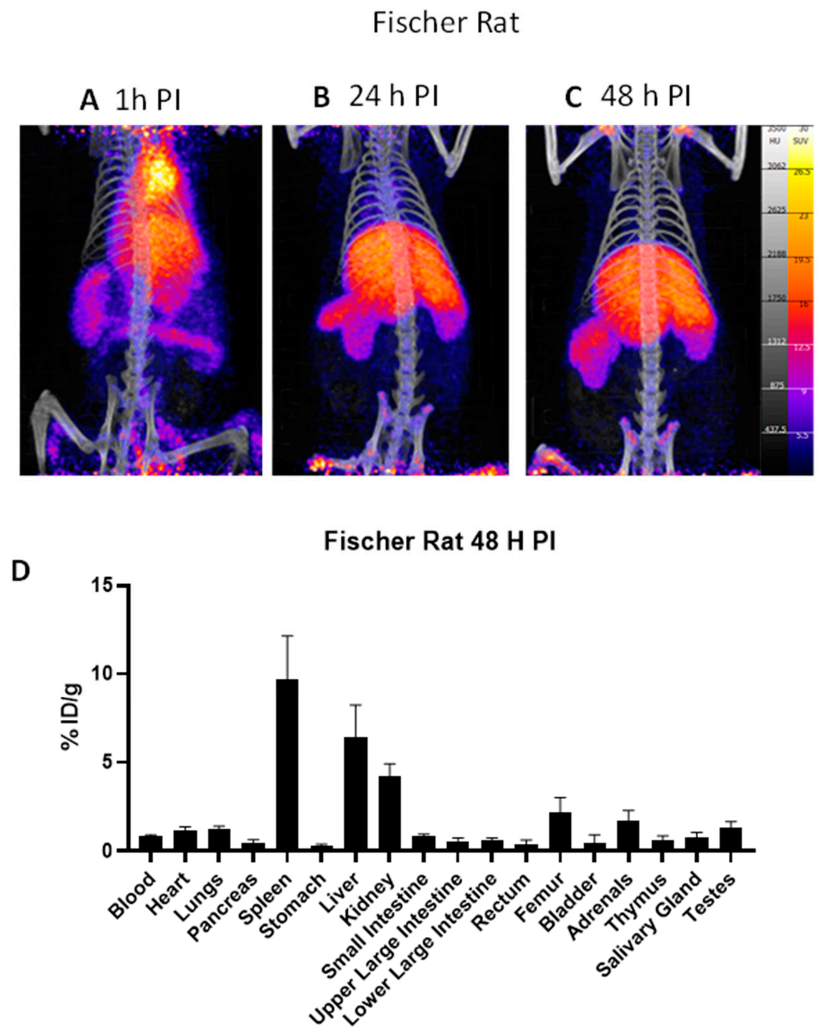

Figure S5: PET/CT imaging and biodistribution in Fischer rats shows similar spleen uptake to mice. PET/CT images at 1 h (**A**), 24 h (**B**), and 48 h (**C**) PI shows the distribution of ^{89}Zr-hAnnA1-C. Biodistribution at 48 h (**D**).

The phenomenon of high splenic uptake of hAnnA1-C was apparent in rats as well (Figure S5). In PET/CT images, the spleen was visible at 1 h PI and continued to become more clear at the 24 h and 48 h timepoints (Figure S5C,D). Biodistribution data at 48 h shows splenic uptake of 9.7 ± 2.4 %ID/g (Figure S5D).
